# Effects of Cigarette Smoking on Resting-State Functional Connectivity of the Nucleus Basalis of Meynert in Mild Cognitive Impairment

**DOI:** 10.3389/fnagi.2021.755630

**Published:** 2021-11-18

**Authors:** Tiantian Qiu, Qingze Zeng, Xiao Luo, Tongcheng Xu, Zhujing Shen, Xiaopei Xu, Chao Wang, Kaicheng Li, Peiyu Huang, Xiaodong Li, Fei Xie, Shouping Dai, Minming Zhang

**Affiliations:** ^1^Department of Radiology, Linyi People’s Hospital, Linyi, China; ^2^Department of Radiology, The Second Affiliated Hospital of Zhejiang University School of Medicine, Hangzhou, China; ^3^Department of Equipment and Medical Engineering, Linyi People’s Hospital, Linyi, China

**Keywords:** mild cognitive impairment, smoking, cholinergic dysfunction, nucleus basalis of Meynert, resting-state functional connectivity

## Abstract

**Background:** Mild cognitive impairment (MCI) is the prodromal phase of Alzheimer’s disease (AD) and has a high risk of progression to AD. Cigarette smoking is one of the important modifiable risk factors in AD progression. Cholinergic dysfunction, especially the nucleus basalis of Meynert (NBM), is the converging target connecting smoking and AD. However, how cigarette smoking affects NBM connectivity in MCI remains unclear.

**Objective:** This study aimed to evaluate the interaction effects of condition (non-smoking vs. smoking) and diagnosis [cognitively normal (CN) vs. MCI] based on the resting-state functional connectivity (rsFC) of the NBM.

**Methods:** After propensity score matching, we included 86 non-smoking CN, 44 smoking CN, 62 non-smoking MCI, and 32 smoking MCI. All subjects underwent structural and functional magnetic resonance imaging scans and neuropsychological tests. The seed-based rsFC of the NBM with the whole-brain voxel was calculated. Furthermore, the mixed effect analysis was performed to explore the interaction effects between condition and diagnosis on rsFC of the NBM.

**Results:** The interaction effects of condition × diagnosis on rsFC of the NBM were observed in the bilateral prefrontal cortex (PFC), bilateral supplementary motor area (SMA), and right precuneus/middle occipital gyrus (MOG). Specifically, the smoking CN showed decreased rsFC between left NBM and PFC and increased rsFC between left NBM and SMA compared with non-smoking CN and smoking MCI. The smoking MCI showed reduced rsFC between right NBM and precuneus/MOG compared with non-smoking MCI. Additionally, rsFC between the NBM and SMA showed a significant negative correlation with Wechsler Memory Scale-Logical Memory (WMS-LM) immediate recall in smoking CN (*r* = −0.321, *p* = 0.041).

**Conclusion:** Our findings indicate that chronic nicotine exposure through smoking may lead to functional connectivity disruption between the NBM and precuneus in MCI patients. The distinct alteration patterns on NBM connectivity in CN smokers and MCI smokers suggest that cigarette smoking has different influences on normal and impaired cognition.

## Introduction

Alzheimer’s disease (AD) is the most common type of dementia in the elderly people, accompanied by progressive and irreversible cognitive decline, affecting over 50 million people worldwide (Prince et al., 2015). Mild cognitive impairment (MCI) refers to the symptomatic pre-dementia phase of AD. Numerous longitudinal studies have reported that MCI is associated with a high risk of progression to AD (Larrieu et al., 2002). However, there is currently no effective treatment to reverse the course of AD. Exploring the early cerebral functional changes in patients with MCI could provide objective bases for guiding clinical intervention and treatment.

Human neuropathological studies showed that the degeneration of cholinergic systems is one of the earliest pathophysiological events in AD (Mesulam et al., 2004; Geula et al., 2008). The cholinergic system mediates a wide range of cognitive functions, including memory, learning, attention, and other high-order brain functions (Hampel et al., 2018). As the most important component of the basal forebrain cholinergic system, the nucleus basalis of Meynert (NBM) provides the primary sources of cholinergic innervation to the limbic and neocortical regions (Gratwicke et al., 2013; Liu et al., 2015). Early postmortem studies of AD patients have emphasized a profound loss of cholinergic neurons, particularly the NBM (Whitehouse et al., 1981, 1982). More importantly, cholinergic dysfunction in the NBM is highly associated with the neurofibrillary tangle, which is the hallmark of AD neuropathology (Mesulam et al., 2004; Mesulam, 2013).

Cigarette smoking is one of the important modifiable risk factors in AD progression (Durazzo et al., 2014; Kivipelto et al., 2018; Johnson et al., 2021). Heavy smoking is associated with a greater than 100% increase in the risk of AD after two decades of exposure (Rusanen et al., 2011). Besides the negative effects on the cerebrovascular burden, cigarette smoking also affects cholinergic functions by its action on nicotinic acetylcholine receptors (nAChRs). For example, chronic nicotine exposure could cause the upregulation of nAChRs or the loss of nicotine-sensitive nAChRs functional activity (Mehta et al., 2012). In addition, smoking-related oxidative stress could also facilitate the extracellular fibrillar β-amyloid (Aβ) aggregation and abnormal tau phosphorylation (Ho et al., 2012; Moreno-Gonzalez et al., 2013), which are the neuropathological hallmarks of AD. Furthermore, Aβ accumulation shows strong co-localization at nAChRs, causing the amyloid-mediated degeneration of the cholinergic system (Buckingham et al., 2009). Thus, cigarette smoking might induce cognitive decline by cholinergic systems, and the NBM could be an important target.

Structural and functional magnetic resonance imaging (MRI) could evaluate the atrophy and functional activity alterations of the NBM *in vivo*. In cognitively healthy individuals, past or current smoking was significantly associated with smaller NBM volume (Teipel et al., 2016). In addition, reduced volume in the NBM was also observed in MCI patients, especially the smokers, and atrophy was more extensive and included the whole basal forebrain in AD patients (Grothe et al., 2012; Kilimann et al., 2014; Brueggen et al., 2015). Numerous functional MRI studies have provided consistent evidence that functional connectivity (FC) disruption precedes structural atrophy and reflect the underlying pathophysiological alterations (Pievani et al., 2011). Based on the resting-state functional MRI (rsfMRI), the NBM showed decreased FC with the posterior cingulate cortex, precuneus, and cuneus in smokers as compared with non-smokers (Zhang et al., 2017). Few studies also found decreased FC of the NBM to insula, precuneus, and lingual gyrus in MCI patients when compared with healthy control (Li et al., 2017; Xu et al., 2021). However, how cigarette smoking affects the FC alterations of the NBM in MCI patients remains unclear.

In this study, we aimed to investigate the smoking effects on the FC alterations of the NBM in patients with MCI. Based on previous work, we hypothesized that the smoking MCI group might have more serious FC disruption of the NBM than the non-smoking MCI group, especially in regions related to nicotine addiction or memory.

## Materials and Methods

### Alzheimer’s Disease Neuroimaging Initiative

The data set used in this study was obtained from the Alzheimer’s Disease Neuroimaging Initiative (ADNI) database^1^. The ADNI was launched in 2003 by the National Institute on Aging (NIA), the National Institute of Biomedical Imaging and Bioengineering (NIBIB), the Food and Drug Administration (FDA), private pharmaceutical companies, and non-profit organizations, as a $60 million 5-year public–private partnership. The primary goal of ADNI is to test whether serial MRI, positron emission tomography (PET), other biological markers, and clinical and neuropsychological assessment can be combined to measure the progression of MCI and early AD. Determination of sensitive and specific markers of very early AD progression is intended to aid researchers and clinicians in developing new treatments and monitor their effectiveness, as well as lessen the time and cost of clinical trials.

### Participants

The Institutional Review Board approved the study of all the participating institutions. Informed written consent was obtained from all participants at each site. Based on the ADNI three databases, we identified 378 right-handed CN subjects and 182 MCI patients with complete neuropsychological evaluations, structural MRI, and rsfMRI scans. We downloaded data from the ADNI database before March 2021. The criteria for MCI in ADNI were: (1) subjective memory complaints, either self-reported, reported by a study partner, or reported by a clinician; (2) objective memory loss defined as scoring below education adjusted cutoff score on delayed recall of the Wechsler Memory Scale-Logical Memory (WMS-LM) test; (3) a global Clinical Dementia Rating (CDR) score of 0.5; (4) a Mini-Mental State Examination (MMSE) score of equal to, or higher than, 24 out of 30; (5) general cognitive and functional performance sufficiently preserved such that a diagnosis of dementia could not be made by the site physician at the time of screening; and (6) no signs of depression (Geriatric Depression Scale, GDS < 6). Criteria for CN were defined as an MMSE score equal to or higher than 24 out of 30, a CDR score of 0, having no report of any cognition complaint, and no signs of depression. Then, we classified MCI and CN subjects into non-smoking and smoking subgroups separately based on self-reported smoking history. Non-smoking subgroup included participants who reported that they never smoked cigarettes during their lifetime, and smoking subgroup included participants who reported any history of smoking (Supplementary Table 1). After screening, 49 participants (including 29 non-smoking CN, 1 smoking CN, and 19 non-smoking MCI) were excluded for excessive head motion (details later). Finally, 304 non-smoking CN, 44 smoking CN, 130 non-smoking MCI, and 33 smoking MCI subjects entered subsequent analyses.

### Demographics and Neuropsychological Data

Demographics, including age, sex, education level, hypertension, diabetes mellitus, and hypercholesterolemia, were assessed. Hypertension was defined as systolic blood pressure >140 mm Hg, diastolic blood pressure >90 mm Hg, medical history of hypertension, or treatment with antihypertensive medication. Diabetes mellitus was defined as random blood glucose ≥11 mmol/dL, medical history of diabetes mellitus, or treatment with glucose-lowering medication. Hypercholesterolemia was defined as random blood cholesterol ≥11 mmol/L, medical history of hypercholesterolemia, or treatment with lipid-lowering medication. Neuropsychological tests in different cognitive domains, such as memory (WMS-LM, immediate and delayed recall), attention [trail-making test, Part A (TMT-A)], execution [trail-making test, Part B (TMT-B)], and language [semantic verbal fluency (SVF)], were included. The demographics and neuropsychological performance of all subjects are listed in Supplementary Table 2.

### Imaging Acquisition

All subjects were scanned using a 3.0-Tesla Philips MRI scanner. The structural images were acquired using a 3D magnetization prepared rapid gradient echo (MPRAGE) T1-weighted sequence with the following parameters: repetition time (TR) = 2,300 ms; echo time (TE) = 2.98 ms; inversion time (TI) = 900 ms; 170 sagittal slices; within plane FOV = 256 mm × 240 mm; voxel size = 1 mm × 1 mm × 1 mm; flip angle = 9°; bandwidth = 240 Hz/pix. The rsfMRI images were obtained using an echo-planar imaging sequence with the following parameters: 197 time points; TR = 3,000 ms; TE = 30 ms; slice thickness = 3.39 mm; spatial resolution = 3.39 mm × 3.39 mm × 3.39 mm; flip angle = 90°; and matrix = 64 × 64.

### Imaging Data Preprocessing

We preprocessed rsfMRI data using the Data Processing & Analysis for Brain Imaging (DPABI^2^) (Yan et al., 2016) with Statistical Parametric Mapping software (SPM12^3^) on the MATLAB platform (MathWorks, Natick, MA, United States). The first 10 time points of rsfMRI data were discarded due to the instability of the initial MRI signal and the adaptation of subjects to the scanning noise. The remaining 187 images were corrected for timing differences between each slice and head motion (six-parameter rigid body). Subjects with more than 3.0 mm maximum displacement in any of the *x*, *y*, or *z* directions or 3.0° of any angular motion were discarded (including 29 non-smoking CN, 1 smoking CN, and 19 non-smoking MCI). Subsequently, rsfMRI images were spatially normalized to the standard EPI template and resampled into 3 mm × 3 mm × 3 mm. The rsfMRI images were spatially smoothed with a Gaussian kernel of 6 mm × 6 mm × 6 mm full width at half maximum. Finally, linear trends and temporally filter (0.01 Hz < f < 0.08 Hz) were performed. Nuisance covariate regression was performed to minimize physiological noise using the Friston-24 head motion parameters (6 head motion parameters, 6 head motion parameters from the previous time point, and the 12 corresponding squared items), as well as white matter (WM) signal and corticospinal fluid (CSF) signal. In addition, the framewise displacement (FD) Jenkinson value of each subject was calculated to correct for the head motion artifacts.

The T1-weighted images were preprocessed and analyzed using the Computational Anatomy Toolbox (CAT12^4^) and SPM12. The images were bias-corrected, tissue-classified [gray matter (GM), WM, and CSF], and registered using linear (12 parameter affine) and non-linear transformations (warping) within the CAT12 default preprocessing pipeline.

### Seed-Based Resting-State Functional Connectivity Analysis

The seed NBM was identified using a probabilistic anatomical map from the SPM Anatomy Toolbox, which was microscopically delineated, 3D reconstructed, and warped to the reference space of the Montreal Neurological Institute (MNI) brain from 10 postmortem human brains (Zaborszky et al., 2008). The NBM, which is the largest of the cholinergic cell clusters constituting the basal forebrain, provides the primary source of cholinergic innervation to the cortex (Gratwicke et al., 2013; Liu et al., 2015). Dynamic brain connectome (DynamicBC) analysis toolbox^5^ (Liao et al., 2014) was used to create individual subject seed-to-voxel connectivity maps. First, each mask was resampled to the dimension of our normalized functional image with 3 × 3 × 3 voxel size for seed-based resting-state functional connectivity (rsFC) analyses. Next, the rsFC maps were generated by calculating the Pearson’s correlation between the time course of the NBM and whole brain areas. Finally, the resulting rsFC maps were transformed to Z maps using Fisher’s Z transformation.

### Propensity Score Matching

Propensity score matching (PSM) implanted in Statistical Product and Service Solutions (SPSS version 26) was performed to balance the differences in demographic features between non-smoking and smoking subgroups in CN and MCI and to reduce the bias due to confounding factors. A propensity score was estimated using multiple logistic regression analysis based on the following covariates: age, sex, and education level. A 1:2 matching was used to pair participants with smoking CN and smoking MCI, respectively. Significant testing and standardized difference (*d*) were applied to assess the balance of covariates before and after PSM. Through PSM, 86 non-smoking CN, 44 smoking CN, 62 non-smoking MCI, and 32 smoking MCI were selected from the initial population. The distribution of propensity scores was relatively balanced after matching (Supplementary Figure 1). There was no significant difference of the key covariates between the smoking and non-smoking groups in both CN and MCI, and the *d* values were acceptable (Supplementary Table 3), suggesting balanced demographic features in CN and MCI subgroups.

### Magnetic Resonance Imaging Features of Small Vessel Disease

We rated the total MRI burden of small vessel disease (SVD) on an ordinal scale from 0 to 4, by counting the presence of each of the four MRI features of SVD, including white matter hyperintensity (WMH), lacunes, cerebral microbleeds (CMBs), and perivascular spaces (PVS) (Staals et al., 2014). The presence of WMH was defined as either confluent deep WMH (Fazekas score 2 or 3) or irregular periventricular WMH extending into the deep WM (Fazekas score 3) on T2 fluid-attenuated inversion recovery (FLAIR) (1 point if present). The presence of lacunes and CMBs was defined as the presence of one or more lacunes on T2 FLAIR (1 point if present) or any CMB on T2^∗^-weighted gradient echo images (1 point if present). The presence of PVS was counted if there were moderate to severe (grades 2–4) PVS in the basal ganglia on T1-weighted axial images (1 point if present) (Zhu et al., 2010). The detailed SVD scores are listed in Supplementary Table 4.

### Statistical Analysis

The statistical analyses of demographics and neuropsychological data were performed using IBM SPSS 26.0 statistical software. We performed a group-level analysis using a one-way ANOVA for continuous variables. If group-level test results were significant, *post hoc* pairwise comparisons were performed (Bonferroni’s multiple comparison correction in parametric tests and Dunn’s multiple comparison tests in non-parametric tests). Binary data, such as sex, hypertension, diabetes mellitus, and hypercholesterolemia, were compared between groups using a chi-square test.

The statistical analyses of the rsFC of the NBM were performed using the DPABI toolbox (Yan et al., 2016). Specifically, we performed a 2 × 2 mixed effect analysis to explore the main effects of condition (non-smoking vs. smoking) and diagnosis (CN vs. MCI), as well as the interaction effects of condition × diagnosis. Age, sex, education level, and head motion (FD value) were used as covariates. To control the effect of cortical atrophy on the functional analysis, normalized modulated (with the volumetric information encoded) GM maps were used as covariate images. The threshold was set to the voxel level at *p* < 0.005 and the cluster level at *p* < 0.05 after Gaussian random field (GRF) correction. The interaction effects between condition and diagnosis before PSM are listed in Supplementary Table 5 and Figure 2. In addition, we also evaluated the interaction effects after controlling for SVD scores (Supplementary Figure 3). To further understand how condition and diagnosis interacted on the rsFC of the NBM, we extracted the mean rsFC values from the interaction regions and further performed *post hoc* pairwise comparisons (*p* < 0.05, Bonferroni’s correction). Finally, partial correlation analysis was performed to investigate the correlation between the mean rsFC values of interaction regions and neuropsychological scores with age, sex, and education level as covariates (*p* < 0.05).

## Results

### Demographics and Neuropsychological Data

After PSM, a total of 86 non-smoking CN, 44 smoking CN, 62 non-smoking MCI, and 32 smoking MCI were included. The demographics and neuropsychological performance are summarized in Table 1. There were no significant differences between subgroups in age, sex, education level, hypertension, diabetes mellitus, and hypercholesterolemia (*p* > 0.05). The MCI subgroups (non-smoking and smoking) showed significantly poor neuropsychological performance on memory, attention, execution, and language compared with CN subgroups (*p* < 0.05). The smoking subgroups (CN and MCI) showed no significant differences in neuropsychological performance compared with corresponding non-smoking subgroups (*p* > 0.05).

**TABLE 1:** The demographics and neuropsychological data.

Variables	Non-smoking CN	Smoking CN	Non-smoking MCI	Smoking MCI	*F*/χ^2^	*p*
	(*n* = 86)	(*n* = 44)	(*n* = 62)	(*n* = 32)		
Age (years)	75.14 ± 7.85	75.83 ± 7.64	76.19 ± 6.83	76.19 ± 7.27	0.30	0.824
Sex (F:M)	40:46	21:23	22:40	12:20	2.64	0.451
Education (years)	16.16 ± 2.34	16.27 ± 2.61	16.13 ± 2.47	16.03 ± 1.98	0.07	0.977
Hypertension, *n* (%)	42 (48.8)	21 (47.7)	29 (46.8)	19 (59.4)	1.50	0.682
Diabetes mellitus, *n* (%)	4 (4.7)	1 (2.3)	3 (4.8)	0 (0.0)	1.98	0.576
Hypercholesterolemia, *n* (%)	49 (57.0)	22 (50.00)	30 (48.4)	19 (59.4)	1.73	0.630
**Memory**						
WMS-LM immediate recall	14.21 ± 3.94	15.23 ± 3.36	10.47 ± 3.52	11.66 ± 4.83	17.85	<0.001^abcd^
WMS-LM delayed recall	12.87 ± 4.16	14.20 ± 3.62	8.24 ± 3.97	9.69 ± 4.64	24.92	<0.001^abcd^
Attention						
TMT-A	32.15 ± 9.92	29.84 ± 6.69	35.02 ± 13.19	37.84 ± 13.11	4.14	0.007^d^
Execution						
TMT-B	83.23 ± 41.46	70.50 ± 27.23	99.56 ± 58.41	104.47 ± 55.76	4.92	0.002^cd^
**Language**						
SVF (animal)	20.77 ± 5.01	21.32 ± 5.87	19.02 ± 4.84	18.56 ± 5.28	3.13	0.027^abcd^
Head motion (FD value)	0.11 ± 0.07	0.12 ± 0.07	0.11 ± 0.06	0.13 ± 0.07	0.91	0.435

*Values are expressed as mean ± SD, number of participants.*

*CN, cognitively normal; MCI, mild cognitive impairment; WMS-LM, Wechsler Memory Scale-Logical Memory; TMT, trail-making test; SVF, semantic verbal fluency; FD, framewise displacement.*

*^*a*–*d*^*Post hoc* analysis further revealed the source of ANOVA difference (^*a*^non-smoking CN vs. non-smoking MCI. ^*b*^Non-smoking CN vs. smoking MCI. ^*c*^Smoking CN vs. non-smoking MCI. ^*d*^Smoking CN vs. smoking MCI) (*p*<0.05, significant difference between the two groups).*

### Condition × Diagnosis Interaction on Resting-State Functional Connectivity of the Nucleus Basalis of Meynert

**FIGURE 1:**
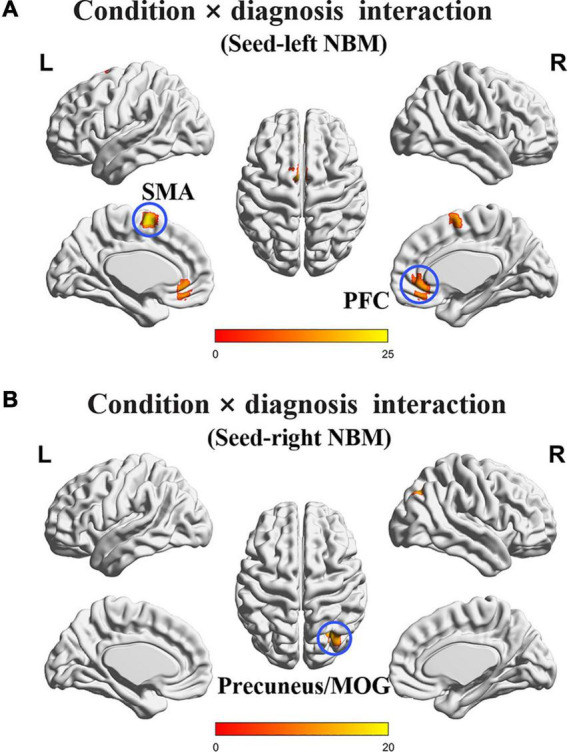
The interaction regions of condition × diagnosis on rsFC of the NBM. **(A)** Based on the seed of left NBM, the interaction regions were primarily located in bilateral PFC and SMA. **(B)** Based on the seed of the right NBM, the interaction region was observed in the right precuneus/MOG. NBM, nucleus basalis of Meynert; PFC, prefrontal cortex; SMA, supplementary motor area; MOG, middle occipital gyrus. The statistical threshold was set at *p* < 0.005 with a cluster level of *p* < 0.05 (two-tailed, GRF corrected).

The interaction effects of condition (non-smoking vs. smoking) × diagnosis (CN vs. MCI) on rsFC of the NBM after PSM are displayed in Figure 1 and Table 2. Based on the seed of left NBM, the regions with interaction effects were primarily located in the bilateral prefrontal cortex (PFC) and bilateral supplementary motor area (SMA). Based on the seed of the right NBM, the interaction region was located in the right precuneus/middle occipital gyrus (MOG) (*p* < 0.005). The main effects of the condition or diagnosis were not detected.

**TABLE 2:** Condition × diagnosis interaction on rsFC of the NBM after PSM.

Seeds	Interaction effect regions	Peak MNI coordinate			Peak intensity	Cluster voxels
		*X*	*Y*	*Z*		
Left NBM	Bilateral PFC	0	36	−9	15.2719	28
Left NBM	Bilateral SMA	−3	0	63	23.7037	24
Right NBM	Right precuneus/MOG	30	−69	33	15.4905	30

*The statistical threshold was set at *p* < 0.005 with a cluster-level of *p* < 0.05 (two-tailed, GRF corrected). NBM, nucleus basalis of Meynert; PFC, prefrontal cortex; SMA, supplementary motor area; MOG, middle occipital gyrus.*

**FIGURE 2:**
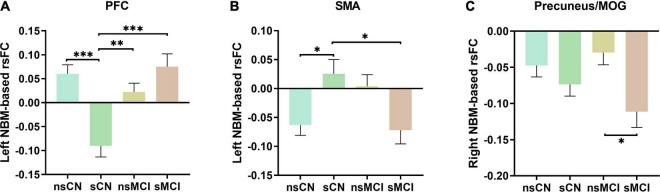
The *post hoc* analysis of interaction regions of rsFC of the NBM. **(A)** The smoking CN (sCN) showed significantly decreased rsFC of left NBM to PFC compared to non-smoking CN (nsCN), non-smoking MCI (nsMCI), and smoking MCI (sMCI). **(B)** The sCN showed increased rsFC of left NBM to SMA compared to nsCN and sMCI. **(C)** The sMCI showed decreased rsFC of right NBM to precuneus/MOG compared with nsMCI. NBM, nucleus basalis of Meynert; PFC, prefrontal cortex; SMA, supplementary motor area; MOG, middle occipital gyrus. ^∗^*p* < 0.05, ^∗∗^*p* < 0.005, ^∗∗∗^*p* < 0.001.

The *post hoc* test revealed the group differences of rsFC values of interaction regions in four subgroups after PSM (Figure 2). Specifically, the smoking CN showed decreased rsFC between left NBM and PFC, and increased rsFC between left NBM and SMA compared with non-smoking CN and smoking MCI. The smoking MCI showed reduced rsFC between right NBM and precuneus/MOG compared with non-smoking MCI.

### Correlation Between Resting-State Functional Connectivity of the Nucleus Basalis of Meynert and Cognition

**FIGURE 3:**
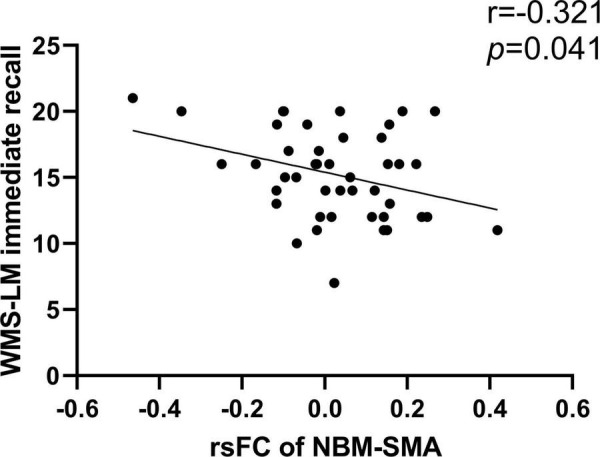
Correlation between rsFC of the NBM and neuropsychological scores. The rsFC of left NBM to SMA was negatively correlated with WMS-LM immediate recall in smoking CN (*r* = –0.321, *p* = 0.041). NBM, nucleus basalis of Meynert; SMA, supplementary motor area; WMS-LM, Wechsler Memory Scale-Logical Memory.

We studied the relationship between rsFC of the NBM and neuropsychological performance in different domains (Table 3). We only found that rsFC of the NBM to the SMA showed a significant negative correlation with memory (WMS-LM immediate recall) in the smoking CN group (*r* = −0.321, *p* = 0.041; Figure 3). After Bonferroni’s correction (*p* < 0.05/15) for multiple comparisons, there was no significant correlation between rsFC of the NBM and neuropsychological scores.

**TABLE 3:** Correlations between rsFC values and neuropsychological scores.

	WMS-LM immediate recall	WMS-LM delayed recall	TMT-A	TMT-B	SVF
**Across all groups**					
Bilateral PFC	0.031	0.013	–0.023	0.073	0.105
Bilateral SMA	–0.051	–0.057	–0.035	–0.055	–0.113
Right precuneus/MOG	–0.013	0.028	0.009	0.077	–0.055
**Smoking CN group**					
Bilateral PFC	–0.134	–0.244	0.031	–0.155	0.011
Bilateral SMA	−0.321*	–0.227	0.054	–0.023	–0.142
Right precuneus/MOG	0.067	0.183	–0.145	0.191	–0.086
**Smoking MCI group**					
Bilateral PFC	0.068	0.013	–0.166	–0.044	0.29
Bilateral SMA	–0.178	–0.231	0.134	0.121	–0.359
Right precuneus/MOG	–0.042	0.094	–0.074	0.008	0.236

Data represent correlation coefficients. WMS-LM, Wechsler Memory Scale-Logical Memory; TMT, trail-making test; SVF, semantic verbal fluency; PFC, prefrontal cortex; SMA, supplementary motor area; MOG, middle occipital gyrus. *p < 0.05, uncorrected.

## Discussion

In this study, we mainly investigated the interaction effects between cigarette smoking and cognitive status based on the rsFC of the NBM. The interaction regions were primarily located in the bilateral PFC, bilateral SMA, and right precuneus/MOG. Furthermore, we observed different alteration patterns on rsFC of the NBM in the smoking CN group and the smoking MCI group. To the best of our knowledge, this is the first study to explore the effects of cigarette smoking on the brain function activity of MCI through the cholinergic pathway.

In the smoking CN group, decreased rsFC between the NBM and PFC was observed compared with the non-smoking CN group. The PFC underlies higher cognitive processes, such as decision-making, working memory, and attention (Passetti et al., 2002; Euston et al., 2012), modulated by nAChRs activation with cholinergic inputs (Dehaene and Changeux, 2011; Bloem et al., 2014a). As the largest cholinergic cell clusters constituting the basal forebrain cholinergic system, the NBM cholinergic neurons mainly project to the PFC (Bloem et al., 2014b; Ballinger et al., 2016). Moreover, the PFC is also an important region of the reward circuit, which plays a key role in addiction (Baker et al., 2003). Thus, our result that showed decreased rsFC between the NBM and PFC could potentially reflect the cholinergic dysfunction by cigarette smoking, which might also explain the working memory and attention decline in healthy smokers observed by previous studies (Chamberlain et al., 2012; Zhang et al., 2016). Previous findings in healthy smokers suggested that smoking was associated with reduced brain volumes (Gallinat et al., 2006; Fritz et al., 2014) or MR spectroscopy markers of neuronal viability (Durazzo et al., 2016; Faulkner et al., 2020), particularly in the PFC. In addition, rsfMRI studies (Janes et al., 2012) also demonstrated that PFC network interactions might serve as biomarkers for nicotine dependence severity and treatment efficacy. Combining our results and previous findings, we speculated that the PFC is the key brain area associated with smoking addiction and cholinergic abnormality.

We also found increased rsFC between the NBM and SMA in the smoking CN group compared with the non-smoking CN group, which is consistent with previous work (Zhang et al., 2017). Many studies have proposed that habitual mechanism plays an important role in addiction (Yalachkov et al., 2009; Jasinska et al., 2014). Individuals with greater nicotine dependence severity had increased engagement of motor preparation circuits, suggesting increased reliance on habitual behaviors (Claus et al., 2013). The SMA is the key brain area associated with automatized behavior and motor planning (Desmurget and Sirigu, 2012). Thus, we inferred that increased rsFC between NBM and SMA might suggest more automatized smoking behaviors for smokers. In previous studies on smoking addiction using rsfMRI, the SMA showed widespread brain connections with reward-related regions, such as the anterior cingulate cortex, insula, and precuneus (Ding and Lee, 2013; Shen et al., 2018; Addicott et al., 2019). Moreover, the increased connectivity of SMA with other regions such as the insula and NBM was associated with the severity of nicotine dependence (Claus et al., 2013; Zhang et al., 2017). Taken together, the SMA could be a vital functional region involved in smoking-related habitual addiction and motor planning.

Additionally, the rsFC of the NBM to SMA in smoking CN showed a negative correlation with WMS-LM immediate recall. The role of SMA in cognitive function such as memory has also been reported in previous studies (Canas et al., 2018; Marvel et al., 2019), and the long-term cigarette smoking could cause difficulty in concentration which in turn induced memory decline (Chamberlain et al., 2012; Zhang et al., 2016). Thus, we speculated that the negative correlation between rsFC of the NBM to SMA and memory could be an early compensatory mechanism to underlying attention-related memory impairment. Our result of decreased rsFC of the NBM to SMA in smoking MCI compared with smoking CN further indicated that this compensatory mechanism might be weakened as cognitive decline progressed.

In the smoking MCI group, the NBM showed decreased rsFC with precuneus/MOG compared with the non-smoking MCI group. The precuneus is an important node of the default-mode network (DMN), a system that contributes to episodic memory retrieval (Raichle et al., 2001). Patients with AD and MCI showed disrupted FC of the precuneus/DMN, which is associated with cognitive decline (Dennis and Thompson, 2014; Eyler et al., 2019). Moreover, the precuneus is also one of the earliest brain regions with Aβ deposition (Long and Holtzman, 2019), which is the hallmark of AD pathology. Longitudinal studies have emphasized that Aβ deposition in the precuneus and other cortical regions comprising the DMN in the pre-dementia stage would sequentially induce regional cortical hypometabolism, accumulation of tau pathology, and hippocampal volume loss (Gordon et al., 2016, 2018; Hanseeuw et al., 2019). However, our study lacked evidence of the correlation between rsFC values of precuneus and Aβ deposition because only a few smoking MCI patients had positive biomarker evidence (i.e., Aβ+). Further studies with Aβ+ smoking MCI patients are needed to verify the role of the precuneus in addiction and cognition. In addition, as for smoking addiction, the precuneus is also recognized as an important contributor to cue reactivity in smokers, through its role in attentional bias toward smoking cues (Engelmann et al., 2012; Courtney et al., 2014). The abnormal brain activity of the precuneus/DMN has also been observed in healthy smokers (Weiland et al., 2015; Chen and Mo, 2017; Zhang et al., 2017; Wilcox et al., 2018). It should be noted that cholinergic systems play a vital role in cognitive resilience and brain plasticity (Drever et al., 2011). Preserved cholinergic forebrain integrity could enable adaptation to the structural degeneration of MCI patients (Ray et al., 2015; Berlot et al., 2021), while smoking could be a risk factor that destroyed this balance. Therefore, we deduced that the precuneus could be an important target of cigarette smoking affecting the cognitive function of MCI through the cholinergic pathway.

### Limitations

Several limitations need to be addressed for this study. First, due to the limited sample size of the ADNI database, the sample sizes of the smoking CN and smoking MCI groups are relatively small compared with the non-smoking groups. In addition, the biomarkers were not included in this analysis because only a few smoking MCI patients had positive biomarker evidence (i.e., Aβ+). Future studies with a larger sample size and biomarker evidence are needed to verify our work. Second, this cross-sectional study is lacking clinical follow-up to make any possible inference between smoking and AD. Thus, longitudinal studies are needed to determine whether rsFC of the NBM alterations in smokers is related to disease progression. Third, our results of the correlation analysis did not survive after multiple comparison corrections. However, this is an explorative study, and our results could partly reflect the effects of cigarette smoking on different cognitions. Finally, smoking history in the ADNI database is defined by subjective self-report from the medical record, including former and current smokers. Many participants lack detailed records such as the number, duration, and status (former or current) of smoking. Future studies may further explore the effects of different smoking degrees or statuses on cognitive functions in MCI because these factors could affect cerebral functional activity.

## Conclusion

Our findings indicate that chronic nicotine exposure through smoking may lead to FC disruption between the NBM and precuneus in MCI patients. Moreover, the distinct alteration patterns on NBM connectivity in CN smokers and MCI smokers suggest that cigarette smoking has different influences on normal and impaired cognition.

## Data Availability Statement

The data used in the preparation of this article were obtained from the Alzheimer’s Disease Neuroimaging Initiative (ADNI) database: http://adni.loni.usc.edu/.

## Ethics Statement

All procedures performed in studies involving human participants were in accordance with the ethical standards of the institutional and/or national research committee and with the 1964 Helsinki declaration and its later amendments or comparable ethical standards. Written informed consent was obtained from all participants and/or authorized representatives and the study partners before any protocol-specific procedures were carried out in the ADNI study.

## Author Contributions

TQ and QZ designed the study and wrote the first draft of the manuscript. XL analyzed the MRI data and wrote the protocol. QZ and XL collected the clinical and MRI data. TX, ZS, XX, CW, KL, PH, XDL, FX, SD, and MZ assisted with the research design and interpretation of results. All authors contributed to the final manuscript and read and approved the final manuscript.

## Conflict of Interest

The authors declare that the research was conducted in the absence of any commercial or financial relationships that could be construed as a potential conflict of interest.

## Publisher’s Note

All claims expressed in this article are solely those of the authors and do not necessarily represent those of their affiliated organizations, or those of the publisher, the editors and the reviewers. Any product that may be evaluated in this article, or claim that may be made by its manufacturer, is not guaranteed or endorsed by the publisher.

## Footnotes

1
http://adni.loni.usc.edu/

2
http://rfmri.org/dpabi

3
http://www.fil.ion.ucl.ac.uk/spm/

4
http://dbm.neuro.uni-jena.de/cat/

5
http://restfmri.net/forum/DynamicBC
