# Supplementary material for: Effects of Cigarette Smoking on Resting-State Functional Connectivity of the Nucleus Basalis of Meynert in Mild Cognitive Impairment
Source: Front Aging Neurosci. 2021 Nov 18;13:755630. doi: 10.3389/fnagi.2021.755630 (PMC8638702; doi:10.3389/fnagi.2021.755630)
Supplement: Supplementary file 1 [file Data_Sheet_1.PDF]

Supplementary Material

Effects of Cigarette Smoking on Resting-State Functional Connectivity of the Nucleus Basalis of Meynert in Mild Cognitive Impairment

Supplementary Table 1A. Smoking information for 44 CN smokers

Subject	Description of smoking information
01	Smoked 1 pack per day for 15 years
02	Smoked 1/2 pack per day for 39 years, and quitted in 1985
03	History of Smoking
04	Smoked 1 pack per day for 42 years, and quitted in 1996
05	Smoked 2 packs per day for 4 years, and quitted in 1966
06	Smoked 1/2 pack per month for 20 years, and quitted in 1966
07	Smoked 1 pack or less per day for 18 years, and quitted 1974
08	Smoked for 26 years
09	Smoked 1 pack per day for 20 years
10	Smoked 1/2 pack per day for 8 years, and quitted in 1974
11	History of Smoking
12	History of Smoking
13	Pipe and cigarette smoking
14	Cigarette smoking
15	Pipe and cigar smoking (4-5 pipes/week, 3-4 cigars/week, - both x 20 years)
16	Smoked 1 pack per day for 8 years, and quitted in 1961
17	Smoked 1 pack per day for 9 years, and quitted in 1954
18	Smoked 1 pack per day for 4 years, and quitted in 1978
19	Smoked 1.5 packs per day for 20 years, and quitted in 1994
20	Smoked 1 pack per day for 40 years
21	Former cigarette smoker
22	Former cigarette smoker
23	Former cigarette smoker, smoked 1 pack per day for 9 years
24	Smoked 1 pack per day for 5 years, and quitted in 1970
25	Smoked for 3 years
26	Smoked 1/2 pack per day for 18 years
27	History of Smoking
28	Former cigarette smoker, smoked 1 pack per day for 20 years
29	Smoked 10 cigarettes per day for 8 years, and quitted in 1992
30	Smoked 1 pack per day for 12 years, and quitted in 1972
31	History of Smoking
32	History of Smoking
33	History of Smoking
34	History of Smoking
35	History of Smoking

36	Smoked cigarettes since his 20's
37	History of Smoking
38	History of Smoking
39	Smoked 5 cigarettes per week
40	Smoked 1/2 pack per day
41	Cigarette smoking
42	Former cigarette smoker
43	Smoked 1-2 cigarettes per day
44	Former cigarette smoker

Supplementary Table 1B. Smoking information for 33 MCI smokers

Subject	Description of smoking information
01	History of Smoking
02	Smoked for 13 years
03	Smoked occasional - not even 1/2 pack per day for 2 years, and quitted in 1944
04	Smoked 2 packs per day for 24 years, and quitted in 2015
05	Smoked for 3 years from 1958 to 1960
06	Smoked 1/2 pack per day for 3 years, and quitted in 1972
07	History of Smoking
08	Smoked 4 pipes per day
09	Smoked 1/2 pack per day for 20 years, and quitted in 1986
10	Former cigarette smoker
11	Cigarette and cigar smoking
12	Smoked 1/10 pack per day for 11 years
13	Smoked 3 packs per day for 10 years, and quitted in 1964
14	Former cigarette smoker
15	Smoked 1/2 pack per day for 22 years, and quitted in 1986
16	Former cigarette smoker, smoked 1 pack per day for 5 years
17	Smoked about 2 packs per day in past
18	Cigarette smoking
19	Former cigarette smoker, smoked 1 cigarette every other week for about 2 years
20	Smoked 1 pack per day
21	Smoked 1 cigar per day
22	Cigarette smoking
23	History of Smoking
24	Smoked 1 packs per day for 40 years
25	Cigarette smoking
26	Smoked 2 packs per day for 23 years
27	Smoked 1.5 packs per day
28	Smoked pipe
29	Former cigarette smoker
30	Cigarette smoking
31	Cigarette smoking

32	Cigarette smoking
33	Cigarette smoking

Supplementary Table 2. The demographic and neuropsychological data.

Variables	Non-smoking CN (n=304)	Smoking CN (n=44)	Non-smoking MCI (n=130)	Smoking MCI (n=33)	F/χ^2	*p*
Age(years)	72.98±7.25	75.83±7.64	74.33±7.87	76.61±7.54	4.09	0.007[b]
Sex(F:M)	183:121	21:23	56:74	12:21	15.62	0.001
Education(years)	16.88±2.33	16.27±2.61	16.71±2.54	15.79±2.40	2.56	0.054
Hypertension, n(%)	118(38.80)	21(47.70)	59(45.40)	20(60.60)	7.04	0.071
Diabetes mellitus, n(%)	7(2.30)	1(2.30)	5(3.80)	0(0.00)	1.84	0.607
Hypercholesterolemia, n(%)	149(49.00)	22(50.00)	73(56.20)	20(60.60)	3.01	0.390
Memory						
WMS-LM immediate recall	14.64±3.61	15.23±3.36	10.62±4.30	11.64±4.76	38.55	< 0.001[abcd]
WMS-LM delayed recall	13.53±3.80	14.20±3.62	8.58±4.37	9.61±4.59	54.94	< 0.001[abcd]
Attention						
TMT-A	31.30±9.51	29.84±6.69	36.15±13.05	37.76±12.91	9.96	< 0.001[abcd]
Execution						
TMT-B	74.85±35.90	70.50±27.23	96.23±51.98	110.39±64.58	14.21	< 0.001[abcd]
Language						
SVF (animal)	21.68±5.12	21.32±5.87	19.02±5.07	18.67±5.23	10.08	< 0.001[ab]
Head Motion (FD value)	0.11±0.07	0.12±0.07	0.11±0.06	0.13±0.07	1.04	0.374

Values are expressed as mean ± standard deviation, number or percentage of participants.

Abbreviation: CN, Cognitively normal; MCI, Mild Cognitive Impairment; WMS-LM, Wechsler memory scale-logical memory; TMT, Trail-Making Test; SVF, Semantic Verbal Fluency; FD, framewise displacement.

[a–d]Post hoc analysis further revealed the source of ANOVA difference ([a] Non-smoking CN vs. Non-smoking MCI; [b] Non-smoking CN vs. Smoking MCI; [c] Smoking CN vs. Non-smoking MCI; [d] Smoking CN vs. Smoking MCI) ($p < 0.05$, significant difference between the two groups)

Supplementary Table 3. Standardized difference (d) before and after PSM in CN and MCI.

	Standardized difference (d) in CN		Standardized difference (d) in MCI	
	Before matching	After matching	Before matching	After matching
Age	0.374	0.088	0.302	-0.037
Male	0.247	-0.022	0.137	-0.032
Education level	-0.231	0.096	-0.384	-0.039

Supplementary Table 4. SVD markers of the four groups.

Variables	Non-smoking CN (n=86)	Smoking CN (n=44)	Non-smoking MCI (n=62)	Smoking MCI (n=32)	F/χ^2	*p*
SVD score					15.13	0.234
0	5(5.8)	0(0.0)	2(3.2)	2(6.3)		
1	41(47.7)	31(70.5)	33(53.2)	23(71.9)		
2	27(31.4)	8(18.2)	15(24.2)	6(18.8)		
3	11(12.8)	5(11.3)	11(17.7)	1(3.1)		
4	2(2.3)	0(0.0)	1(1.6)	0(0.0)		
WMH(1 point)	32(37.2)	10(22.7)	23(37.1)	4(12.5)	9.18	0.027
Lacunes(1 point)	18(20.9)	7(15.9)	13(21.0)	3(9.4)	2.57	0.464
CMBs(1 point)	6(7.0)	1(2.3)	4(6.5)	1(3.1)	1.73	0.63
PVS(1 point)	80(93.0)	44(100.0)	60(96.8)	30(93.8)	3.79	0.285

SVD, small vessel disease; WMH, white matter hyperintensity; CMBs, cerebral microbleeds. PVS, perivascular spaces.
Data presented as number (%) or mean ± standard deviation.

Supplementary Table 5. Condition×diagnosis interaction on rsFC of the NBM before PSM.

Seeds	Interaction effect regions	Peak MNI coordinate			Peak intensity	Cluster voxels
		X	Y	Z		
Left NBM	Bilateral PFC	0	36	-9	15.6291	38
Left NBM	Bilateral SMA	-3	0	63	24.1979	26
Right NBM	Right precuneus/MOG	30	-69	33	15.6381	31
Right NBM	Bilateral calcarine	6	-66	18	14.9852	20

The statistical threshold was set at $p < 0.005$ with a cluster-level of $p < 0.05$ (two-tailed, GRF corrected). NBM, Nucleus Basalis of Meynert; PFC, prefrontal cortex; SMA, supplementary motor area; MOG, middle occipital gyrus.

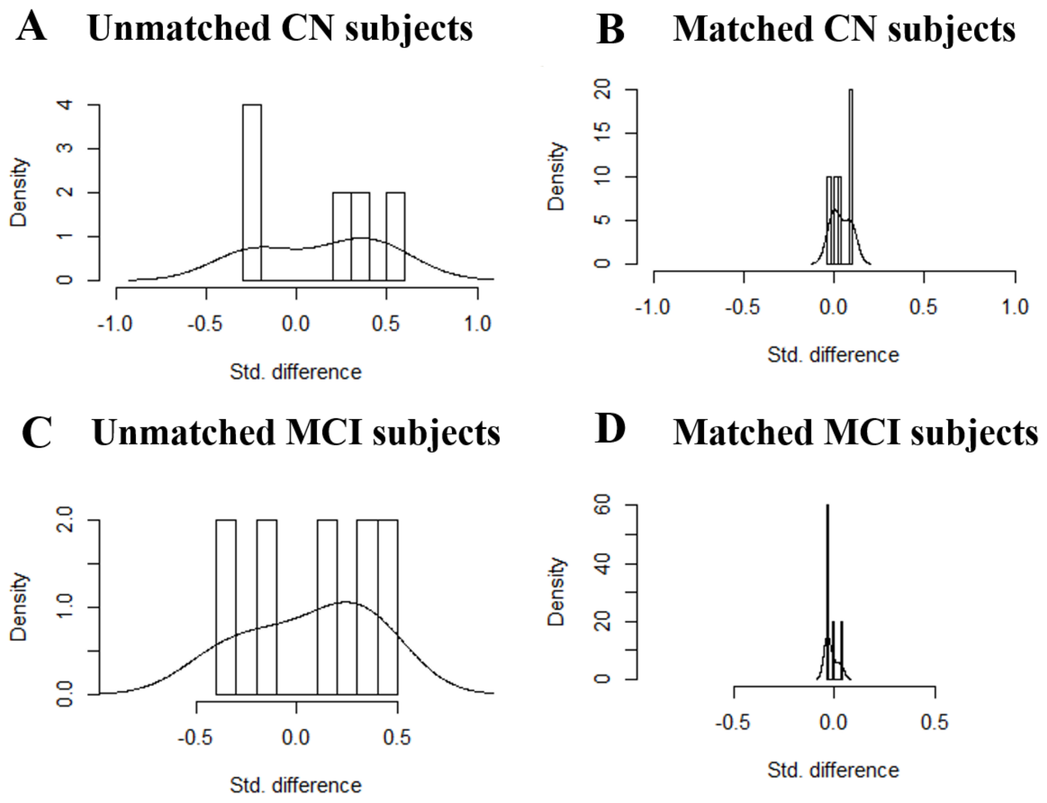

Supplementary Figure 1. Histogram of Standardized difference of CN and MCI subjects before and after PSM.

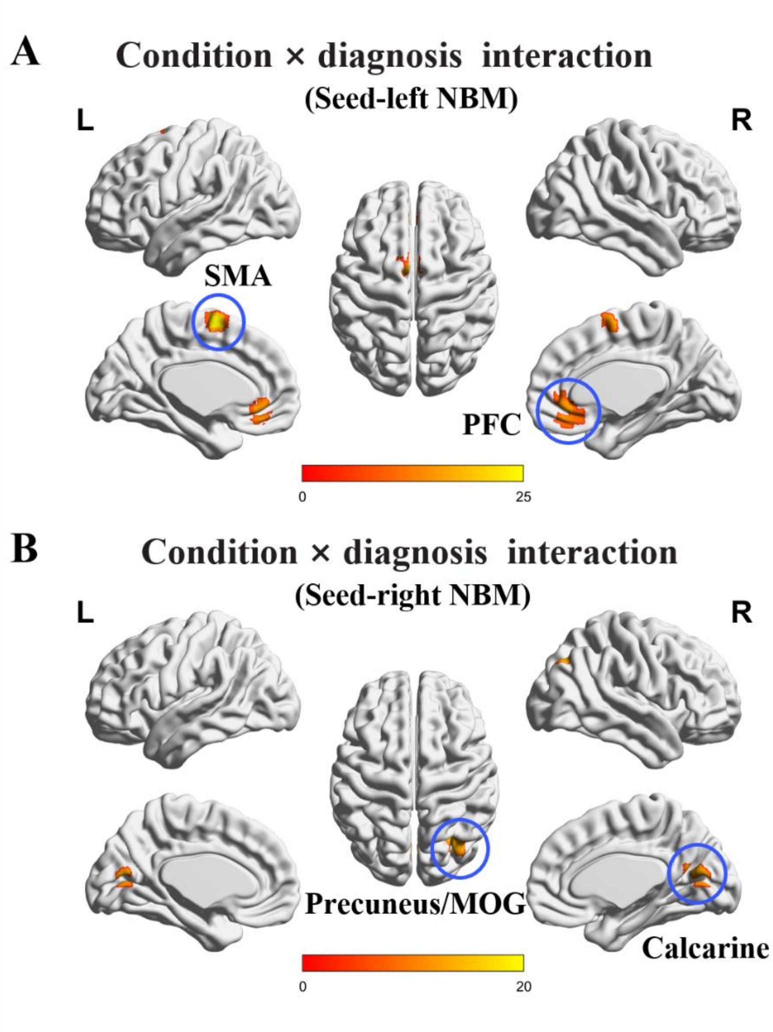

Supplementary Figure 2. The interaction regions of condition × diagnosis on rsFC of the NBM before PSM. (A) Based on the seed of left NBM, the interaction regions were primarily located in the bilateral PFC and SMA. (B) Based on the seed of right NBM, the interaction region was observed in the right precuneus/MOG and bilateral calcarine. NBM, Nucleus Basalis of Meynert; PFC, prefrontal cortex; SMA, supplementary motor area; MOG, middle occipital gyrus. The statistical threshold was set at $p < 0.005$ with a cluster-level of $p < 0.05$ (two-tailed, GRF corrected).

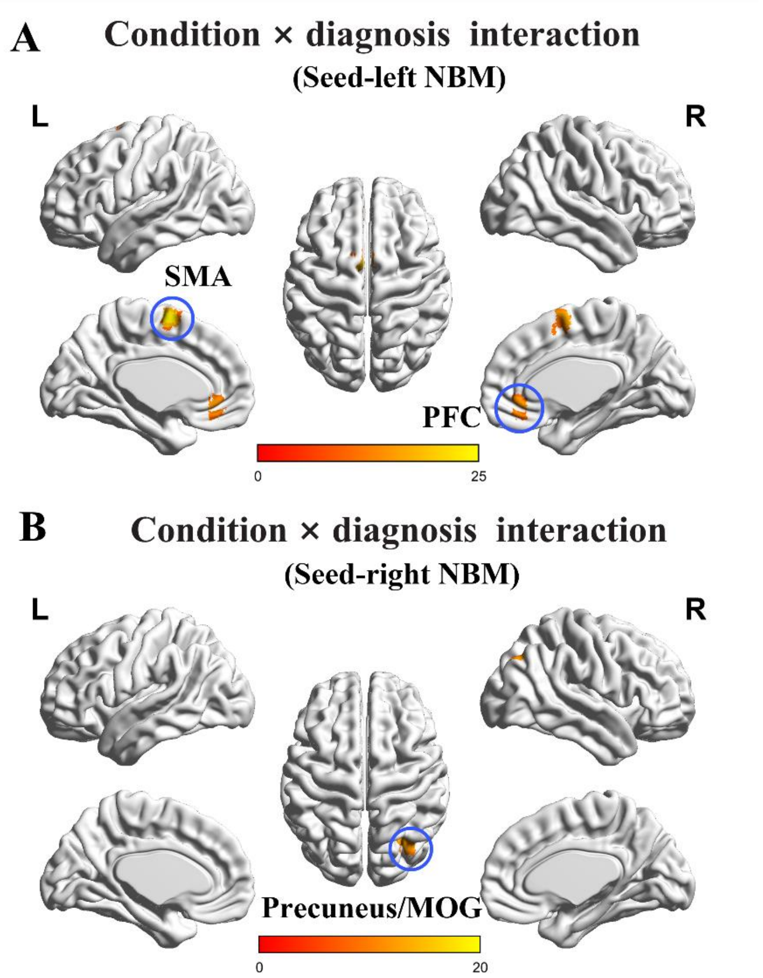

Supplementary Figure 3. The interaction regions of condition × diagnosis on rsFC of the NBM after PSM. (A) Based on the seed of left NBM, the interaction regions were primarily located in bilateral PFC and SMA. (B) Based on the seed of right NBM, the interaction region was observed in right precuneus/MOG. NBM, Nucleus Basalis of Meynert; PFC, prefrontal cortex; SMA, supplementary motor area; MOG, middle occipital gyrus. The statistical threshold was set at $p < 0.005$ with a cluster-level of $p < 0.05$ (two-tailed, GRF corrected) with age, sex, education level, head motion, GM maps, and SVD scores as the covariates.
